# Observation-based estimates of land availability for wind power: a case study for Czechia

**DOI:** 10.1186/s13705-019-0234-z

**Published:** 2019-12-17

**Authors:** Felix Nitsch, Olga Turkovska, Johannes Schmidt

**Affiliations:** 1 Department of Energy Systems Analysis, Institute of Engineering Thermodynamics, German Aerospace Center (DLR), Stuttgart, Germany; 2 Institute for Sustainable Economic Development, University of Natural Resources and Life Sciences, Vienna, Austria

**Keywords:** Renewable energies, Wind power, Expansion scenario, Spatial potential analysis, Land use

## Abstract

**Background:**

The availability of land for the installation of wind power turbines is restricted by numerous factors. Besides climatic conditions, the deployment of wind energy is limited by technical, social, economic, and environmental factors. Typically, assessments of land availability for wind power use legal and technical criteria to estimate the potential for wind power expansion. In contrast, we use observed characteristics of wind power generation sites existing in Austria and Denmark to estimate its potential expansion in Czechia. We combined data on wind turbine locations with data on land use, wind speeds, human impact on land, and nature conservation areas.

**Results:**

Our analysis shows that the density of wind power in Austria is variable, but higher on average (4.79 MW km^−2^) than in Denmark (1.76 MW km^−2^). Austrian wind turbines have been installed in areas where the human impact on land is mostly higher than the Austrian average, while in Denmark, no difference is observed. Regarding the land use composite, the share of agricultural land on sites with wind turbines is on average much higher (86%), while the share of forest is much lower (7%) in both countries. We identified a maximum potential area in Czechia of 543 km^2^ with Austrian and 421 km^2^ with Danish characteristics. When conservatively assuming observed historical power densities, this area translates to 2295 MW and 741 MW of installed wind power capacity, respectively. These results are a magnitude of order lower than the potentials found in existing studies. In a sensitivity analysis, we have examined that the availability of potential sites depends mainly on the population density, the human impact on land, prevailing wind speeds, and the height above sea level.

**Conclusions:**

We estimated available land area for potential wind turbine installations in Czechia using our newly developed methodology based on observed site characteristics of today’s wind power infrastructure in Austria and Denmark. Available land area indicated possible overestimation of wind power capacities proposed in the recent studies on the renewable energy transition. Hence, more rigorous consideration of land availability is required for assessments of potential wind power expansion.

## Background

The transition to energy systems with high shares of renewables requires a significant increase in renewable energy capacities, as has been shown for different world regions [1–5]. Since such a large-scale adoption of renewable energies would lead to new challenges regarding material availability, life cycle assessments have focused on the impacts of wind power plants (WPP) and photovoltaics on the environment [2, 6–9]. Availability of land for the expansion of renewable energies, however, is only a minor [2, 3] or not an issue at all in the recent integrated modeling assessments [4, 5]. In a review of modeling studies, Loftus et al. [10] confirmed that land use and other non-cost barriers such as labor, social acceptability, convenience, and governance constraints of renewable energy scenarios receive little attention in integrated energy system modeling studies. Nevertheless, there is an increasing amount of work assessing land availability for renewable energies, in particular, for wind power. These studies used geographic information systems (GIS) to derive land availability from a set of pre-defined criteria. Ryberg et al. [11] recently reviewed and analyzed the criteria applied in such land availability assessments, showing the inconsistencies between studies. Prevailing approaches among the studies define land as unavailable based on existing legal [12, 13], technical [14–18], or political [19–21] criteria.

This approach potentially produces an overestimation of land availability, as anything not excluded by some criteria is considered to be available for wind power deployment. Additionally, there are probably many factors not being captured, as they are hard to measure by quantitative indicators. Nevertheless, these factors are significant for considering potential WPP allocation. For instance, public opposition against new WPP can interfere with wind expansion scenarios [21], making many estimates too optimistic. As Brewer et al. [22] and Höltinger et al. [21] showed, the potentials of renewable energies can be drastically lower once social acceptance is considered in the assessment. While the opinion of residents close to WPP parks [23] and in economically underdeveloped areas [24] is mainly supportive to wind power technology, this may change into opposition due to the esthetics of wind parks [25] or the perceived characteristics of the implementation procedure for WPP projects, such as fairness [26]. Another factor hardly captured is how the existing density of WPP installations impacts future expansion. The potential density of WPP in modeling studies is mostly considered by determining minimum distances between turbines which are used to account for the reduction of generation due to wake effects [13, 18]. Miller et al. [27], however, found that observed densities are often much lower, possibly driven by technical, economic, and acceptance issues.

Incorporating these aspects into the assessments of WPP potentials is a big challenge as directly measuring social acceptance, and other factors contributing to the reduction of land available for wind power, is hardly possible. In contrast to existing studies, which largely neglect these issues, we therefore use observed deployment levels, densities, and observed site characteristics of WPP in two countries, i.e., Austria (AT) and Denmark (DK), to model potential WPP expansion in a third country, i.e., Czechia (CZ). Observed deployment levels, installation densities, and site characteristics of WPP allow to implicitly consider social acceptance in the land availability exercise. These selection criteria imply that spread and allocation of WPP in studied countries is sufficient for ensuring a certain degree of social acceptance. This approach, of course, assumes that future deployment levels are similar to today’s and may therefore underestimate the land available for WPP generation in the long-term. Nonetheless, we believe that combining traditional land availability analyses with our assessment will give a much more comprehensive view of the potential for deploying wind energy.

Assessments of wind generation potential can be split into land availability, technical, and economic potentials [14]. In the present paper, we focus on the first of the three potentials, i.e., land availability. We see our results as potential inputs to subsequent, more detailed technical, and economic studies. We therefore provide estimates of installable capacity, but not the amount of electricity that may be generated from these turbines as this exceeds the scope of this work.

We use data from European countries with high WPP capacities, where respective data sets on wind power plant locations are available. AT and DK have significant WPP installations and for both countries, spatially explicit data on turbine level is available. CZ is selected as a case study country for assigning Austrian and Danish site characteristics, as it currently has a very low capacity of wind power installed. This paper will therefore add in understanding the characteristics of today’s WPP sites in terms of land use and site specifications and explore the potential land availability for future WPP expansion in CZ based on observed characteristics of existing WPP in AT and DK.

## Methods and data

**Fig. 1:**
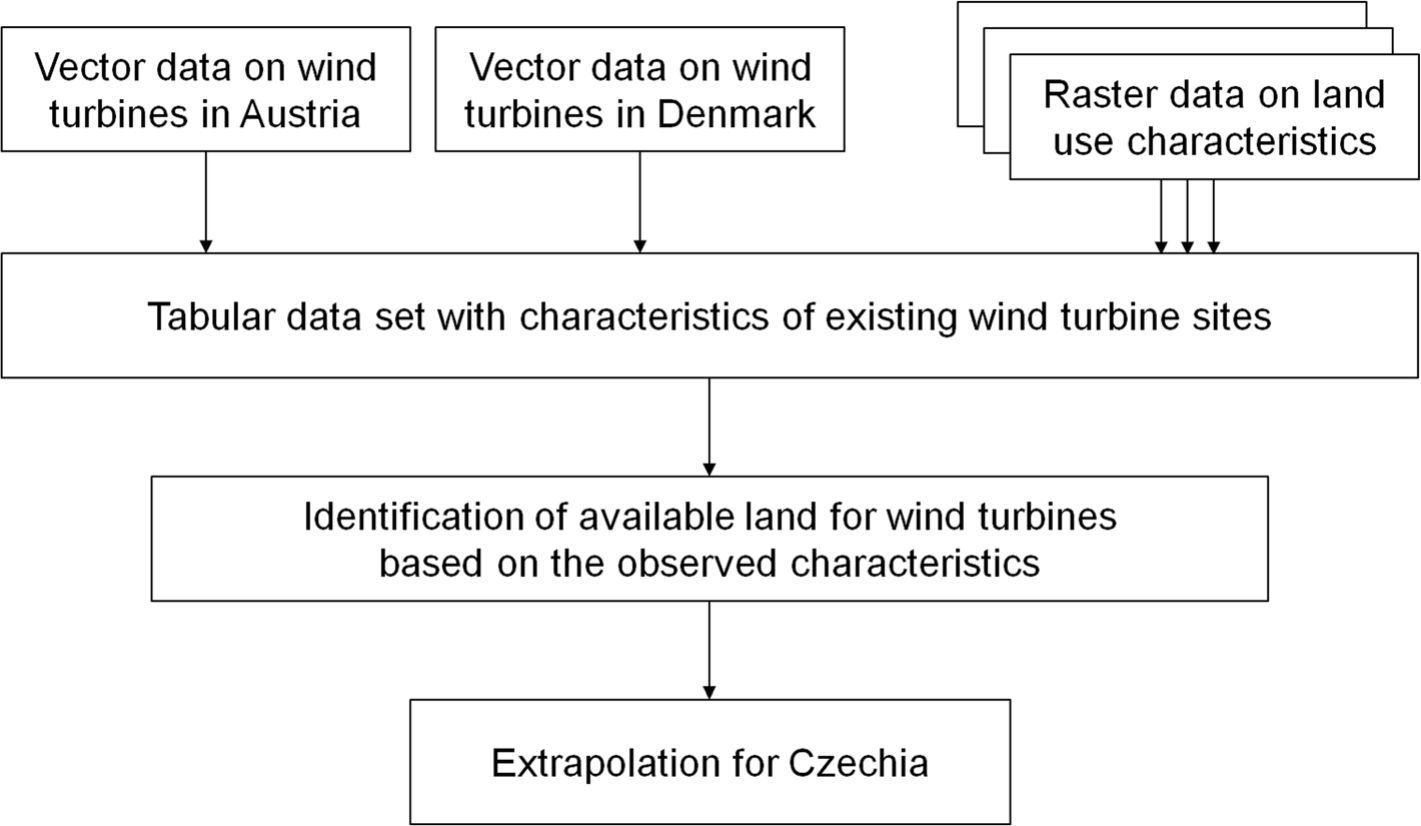
Schematic overview of the methodological approach

Figure 1 illustrates the methodological approach of this study. We produced a new data set at 1-km spatial resolution by aggregating data on existing WPP sites with land- and population-related data sets. We derived wind power capacity per area as well as technical and land characteristics from the aggregated data set. Based on selection criteria derived from observed WPP sites, we identified potential sites for WPP. In this case study, we used AT and DK as reference countries to derive conditions for the potential spatial allocation of WPP in CZ. Our approach, however, can easily be applied to other European countries, as the necessary land-related data sets are available for the whole of Europe. Countries that serve as reference for defining spatial allocation of WPP require spatially highly resolved data for WPP, at best at the level of turbines. To the best of our knowledge, such data sets are unfortunately currently not available for the whole of Europe.

### Data

Table 1 lists all data sets used in this analysis with information on area of application, data type, and spatial resolution. Data on present WPP installations with accurate spatial resolution is critical for our approach. For WPP in DK, we used the Open Power System Data platform which provides open data dedicated to electricity system research [28]. The platform is a widely used tool for modeling and scientific research in energy systems in recent years [37–41]. The full data set for DK included 5615 entries for onshore WPP sites in the database. A total of 1222 small-scale turbines with a total capacity of 14.51 MW were removed from the data set as they are missing location data and therefore not suitable for a spatial analysis. The remaining 4393 entries feature detailed information on location and electrical capacity of WPP (3881.7 MW). Since the Open Power System Data does not contain data on WPP in AT, a data source from the Austrian Wind Energy Association [29] was used instead. The data set features 1071 WPP sites with a total capacity of 2295.5 MW and contains information on location and electrical capacity. Detailed plots on rotor diameter (Figure 7) and hub height (Figure 8) are shown in the Appendix of this paper.

**Table 1:** Data sets for this study

*α*	Name	Description	Area	Source	Format
D1	Open Power System Data	Open source data set featuring information on location, capacity, height, year of installation, etc. of WPP in DK in 2017	DK	Open Power System Data [28]	Vector (point)
D2	Austrian wind power plants	Data set featuring information on location, capacity, year of installation, height, etc. of WPP in AT in 2015	AT	Austrian Wind Energy Association [29]	Vector (point)
D3	Wind speed	Mean wind speed in 100 m and 200 m height	Global	International Renewable Energy Agency [30]	Raster (1 km)
D4	Height above mean sea level	Digital elevation model in 2011	EU	European Environment Agency [31]	Raster (25 m)
D5	Land use	Land use in 2010 according to the LUISA Modeling Platform	EU	Lavalle [32]	Raster (1 km)
D6	Population	Population distribution in 2010 according to the LUISA Modeling Platform	EU	Lavalle and Jacobs Crisioni [33]	Raster (1 km)
D7	National parks	National parks, important wildlife and conservation areas in 2018	AT, CZ, DK	OpenStreetMap [34]	Vector (polygon)
D8	Natura 2000	Natura 2000 conservation areas in 2017	EU	European Environment Agency [35]	Vector (polygon)
D9	Human Footprint Index	Cumulative impact of direct pressures on nature from human activities in 2009	Global	Venter et al. [36]	Raster (1 km)

Prevailing wind speeds are a crucial parameter for the selection of potential WPP sites. The International Renewable Energy Agency [30] publishes the Global Wind Atlas, where annual average wind speeds can be downloaded in raster format [42]. We used two data sets from this source: the average wind speed at 100 m and 200 m height above ground which were both updated in May 2017. The data sets are available at 1-km spatial resolution. Although we did not assess any particular WPP model and a specific hub height, we used these two wind data sets as an indicator for wind resource potential. The data on height above mean sea level was provided by the European Environment Agency [31]. The digital elevation model is available for the years 2000 and 2011 and can be downloaded on the website of the Copernicus Land Monitoring Service. The high spatial resolution of 25 m pixels for all regions makes this data set a powerful source for spatial analysis with vertical accuracy of ± 7 m RMSE according to the European Environment Agency [31]. For data on land use, the Land Use-based Integrated Sustainability Assessment (LUISA) was used. LUISA is a modeling platform used for the ex ante European Commission policy evaluation compiled by Lavalle [32]. It contains pixel values on land use at a spatial resolution of 1 km and is available for open-access download from the Joint Research Centre Data Catalogue [43]. The information on population distribution in the research area was also derived from the LUISA platform [33, 43]. The respective pixel values store information on the population density per square kilometer.

Nature and wildlife conservation have high priorities in the process of wind park planning and operation [44–47]. According to the literature and public opinion, “green” and modern electricity generation should minimize interference with nature. The welfare of birds is of particular concern [48–51]. Additionally, national law often limits the installation of WPP in certain conservation areas. For these reasons, we included data on national parks in our analysis. There are six national parks in AT (Donau-Auen, Gesäuse, Hohe Tauern, Kalkalpen, Neusiedler, see - Seewinkel and Thayatal), five national parks in DK (Vadehavet, Thy, Mols Bjerge, Skjoldungernes Land, and Kongernes Nordsjælland), and four in CZ (Krkonoše, Podyjí, Šumava, and České Švýcarsko). Shape files from the OpenStreetMap [34, 52] were used to identify the areas of national parks in the research area. Furthermore, we integrated Natura 2000 areas into the analysis since they represent important bird protection areas and valuable habitats for many species. There is a data set coordinated by the European Environment Agency and made accessible on the website of the European Environment Agency [35]. Moreover, we used the Human Footprint Index (HFI) developed by Venter et al. [36] in its most recent version of 2009. This raster pixel data set accumulates the impact of human activities on nature by merging data on infrastructure, land use, population, nighttime lights, and waterways. The index ranges from 0 (no human impact) to 50 (highest human impact) and covers most parts of the world making changes of the human impact on land visible.

### Method

First, we performed an aggregation of the observed installed capacity of WPP in the research area to allow a comparison of WPP densities. We generated a custom grid with a pixel size of 1 km for the case study area. The locations of operating WPP were assigned to the respective pixels resulting in a data set which features the aggregated installed capacities in MW km^−2^. As a second step, we merged the remaining spatial data sets D3 to D9 (see Table 1) into one data set. The final complete tabular data set included information on mean wind speeds, height above mean sea level, population density, land use class, HFI, and nature conservation areas. Vector data D7 and D8 were converted into the raster pixel data format in order to be ready for the merging process. When merging, the nearest neighboring raster pixels were identified to match our chosen spatial resolution of 1 km. As a third step, assuming that potential pixels for wind power deployment in CZ have similar characteristics as the ones observed at the current WPP in AT and DK, we identified pixels for potential WPP installations in CZ. We looked at the spread of observed characteristics, i.e., parameters, based on the interquartile range. We estimated a lower threshold *L*_*c*, *d*_ for each selection criterion *P*_*c*, *d*, *i*_ as its first quartile value *Q*_0.25_, where *c* refers to the country (AT, DK), *d* is a data set (D3 … D9), and *i* is a pixel with currently installed WPP (Eq. (1)). This procedure was conducted for AT and DK individually, resulting in two different country-specific lower thresholds for each selection criterion *P*_*c*, *d*, *i*_.

1 $$ {L}_{c,d}={Q}_{0.25}\left({P}_{c,d,i}\right) $$

We defined the upper threshold *U*_*c*, *d*_ in Eq. (2), as the third quartile (*Q*_0.75_) for each selection criterion *P*_*c*, *d*, *i*_.

2 $$ {U}_{c,d}={Q}_{0.75}\left({P}_{c,d,i}\right) $$

Equation (3) describes the filtering condition. *L*_*c*, *d*_ and *U*_*c*, *d*_ are the lower and upper limits defined in Eqs. (1) and (2). All pixels *j* without installed WPP had to meet the condition in order to be identified as eligible for WPP installations.

3 $$ {L}_{c,d}<{P}_{c,d,j}<{U}_{c,d} $$

This conditional filtering method was applied using the attributes D3 to D9, as outlined in Table 1.

The filtering of the data is illustrated here with an example of the selection criterion *P*_AT, landUseAgriculture, i_. This criterion contains the share of land use “agriculture” in AT in all pixels with installed WPP. From all these pixels in AT with WPP installed, the first quartile *Q*_0.25_(*P*_AT, landUseAgriculture, i_) and the third quartile *Q*_0.75_(*P*_AT, landUseAgriculture, i_) were derived from the land use shares. These values determined the lower *L*_AT, landUseAgriculture_ (0.77) and upper bound *U*_AT, landUseAgriculture_ (1.0). All pixels in CZ without any WPP installations *P*_CZ, landUseAgriculture, j_ were filtered according to this criterion, i.e., all pixels where 0.77 < *P*_CZ, landUseAgriculture, j_ < 1.0 are chosen. This process was carried out with all available criteria from Table 1. For the criterion wind speeds, we removed the upper threshold condition as higher average wind speeds would in general not prevent WPP installations. The result is a list of pixels matching all criteria and filtering conditions as described here. Additionally, we calculated a total potential capacity by multiplying the area of identified pixels by the observed mean capacity densities (MW km^− 2^) of pixels with WPP.

We derived potentials for CZ, by applying the AT thresholds derived from the first and third quartile for CZ pixels in scenario S1 and the DK thresholds in scenario S2. Since the minimum observed wind speeds in DK are significantly higher than most of observed wind speeds in CZ, scenario S2 is using the lower boundary condition derived from AT wind speeds. We applied the quartile values from AT sites as threshold for the criterion “height above mean sea level” in scenario S2, because all DK pixels are below the ones in CZ. Consequently, all pixels would be excluded from being available for WPP installation. Our method is sensitive to how the limits in the selection process are defined, e.g., instead of using the lower and upper quartiles, the minimum and maximum in the observed distribution could be used. The impact on results is therefore assessed in a sensitivity analysis.

Our analysis has been performed in Python 3.6 and is available in an open-access github repository [53].

## Results

**Fig. 2:**
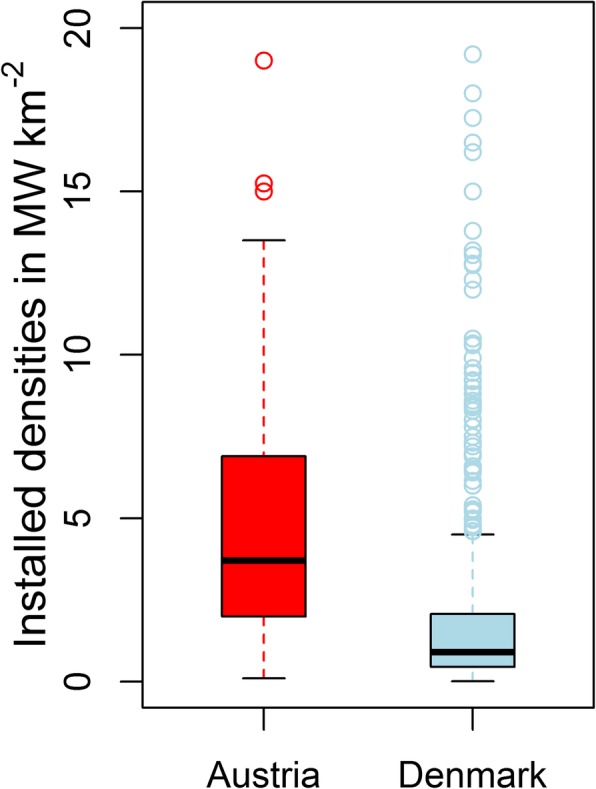
Installed densities of WPP in AT and DK in MW km^−2^

The first part of the analysis shows the installed densities of wind power capacities in AT and DK in Fig. 2. In AT, we observe 479 pixels—out of 83,919 pixels—and in DK 2207 pixels—out of 43,150 pixels—with WPP installed. The mean density is 4.79 MW km^− 2^ for AT and 1.76 MW km^− 2^ for DK. The pixels with the highest densities are almost similar for both countries with 19.00 MW km^− 2^ in AT and 19.20 MW km^− 2^ in DK.

**Fig. 3:**
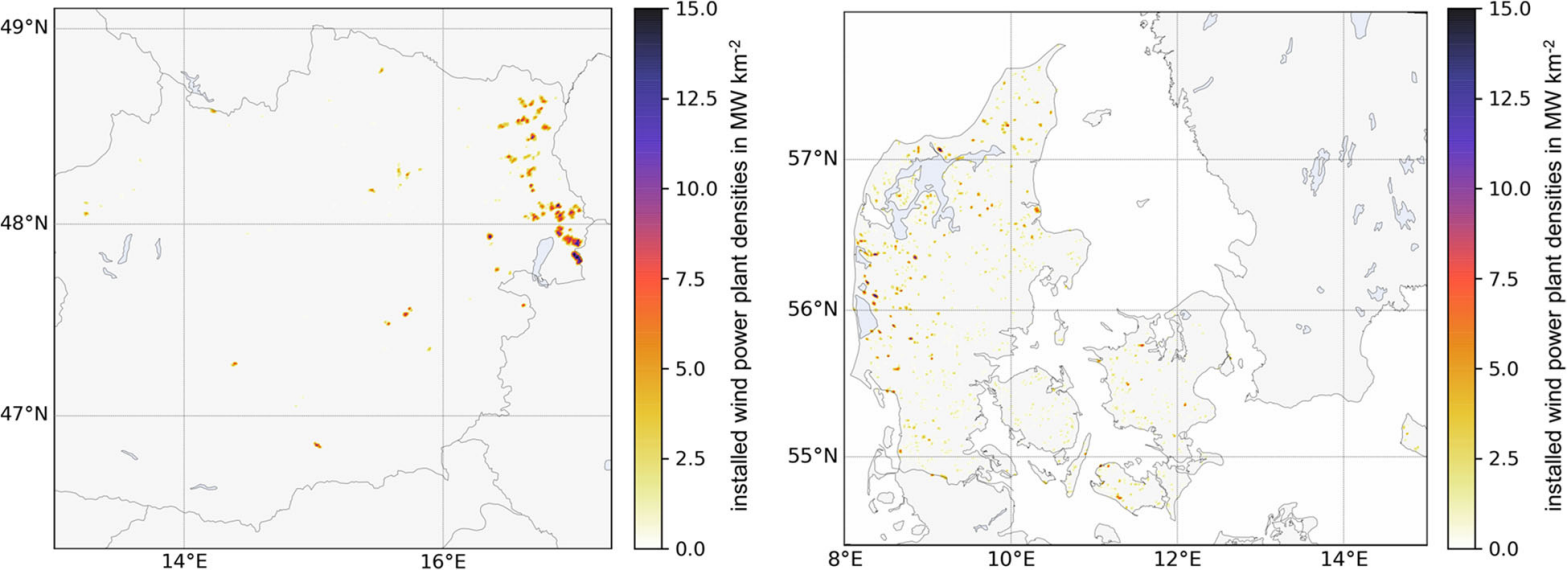
Installed WPP densities in AT (left) and DK (right) shown in MW km^−2^. (There are no WPP installed in the west of Austria; therefore, it is omitted in the map)

The spatial allocation of the WPP is very different for the two countries, as shown in Fig. 3. In AT, there is a clear concentration of WPP in eastern lower AT and northern Burgenland. The Tauernwindpark, which is located in Styria at around 1900 m above sea level with a total capacity of more than 22 MW [54], is the most significant WPP site in the Austrian Alps. The WPP in DK is more evenly distributed over the country. However, we can observe a higher concentration near the coastal areas in the northwest of DK.

### Observed site characteristics

**Fig. 4:**
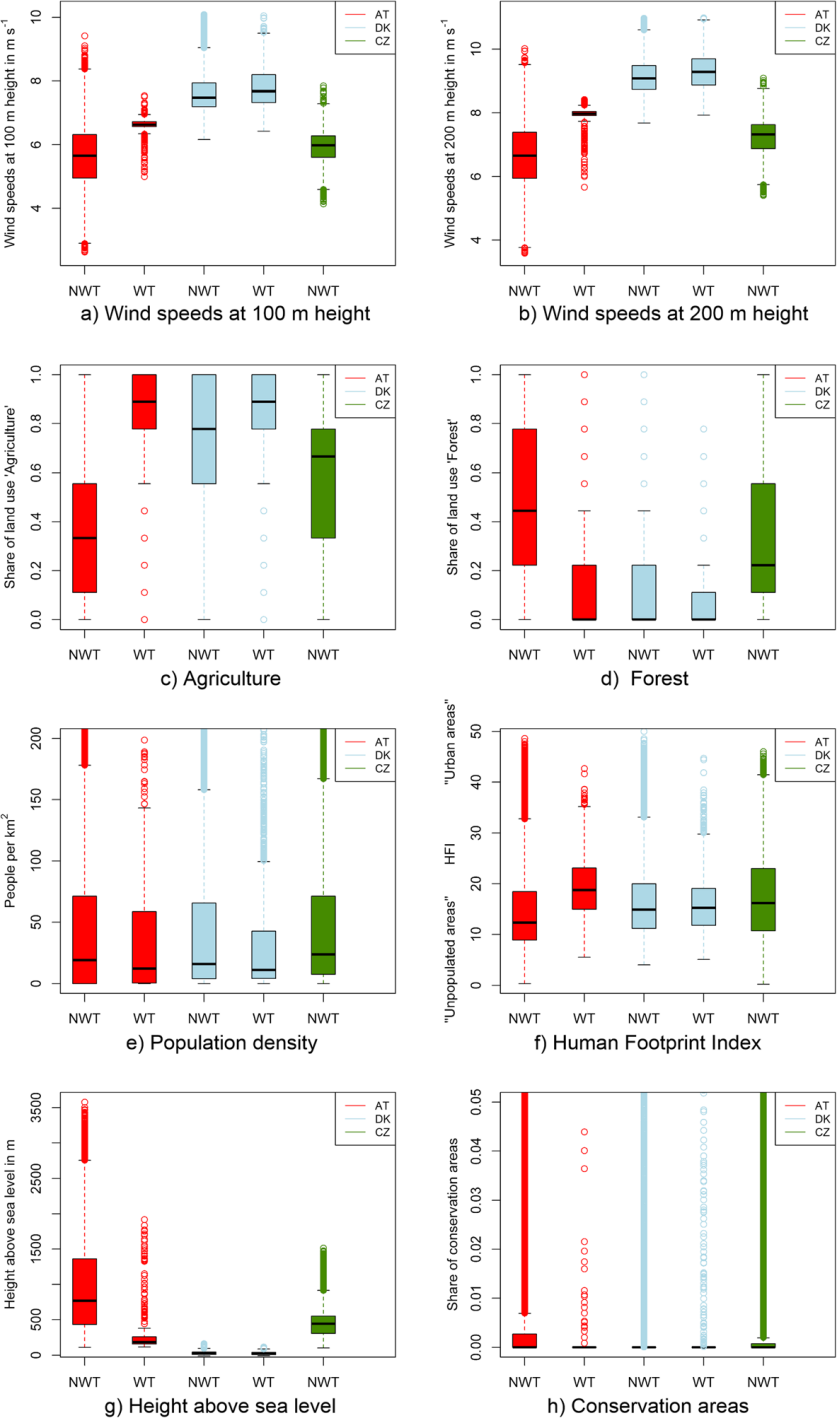
Comparison of site characteristics for pixels without wind turbines (NWT) and with at least one turbine installed (WT)

The box plots in Fig. 4 show the comparison of pixels with no wind turbines (NWT) and with wind turbines (WT) for individual selection criteria in all countries. This allows a comparison of the characteristics of pixels where no use of wind power generation is observed and pixels with WPP deployment. WPP in AT are mainly concentrated in pixels where annual wind speeds at 100 m height above ground are between 5.0 and 7.5 m s^−1^ (Fig. 4a). In DK, observed wind speeds at 100 m height above ground at WPP sites are generally higher than in AT but also with a greater spread in the range from 6.4 to 10.1 m s^−1^. A similar figure shows the plot for annual wind speeds at 200 m height above ground (Fig. 4b), which are overall higher than the wind speeds at 100 m height. In AT, WPP are located in pixels with wind speeds between 5.7 and 8.4 m s^−1^. In DK, the wind speeds are concentrated in a range between 7.9 and 11.0 m s^−1^. We also compared the share of agriculture (Fig. 4c) and forest (Fig. 4d) in the pixels. Generally, the higher the share of agriculture and the lower the share of forest, the higher are the installed capacities at these particular pixels. This can possibly be explained by the fact that forests tend to be more likely in areas which are not as easy to access as agricultural land, therefore increasing construction costs. Also, environmental constraints may be in place in some forests. Most pixels with considerable high shares of WPP have close to zero population density (Fig. 4e). Looking at the first and third quartile, the population density ranges between 0.4 and 58.6 people km^−2^ in AT, and between 4.1 and 42.7 people km^−2^ in DK. Regarding the HFI (Fig. 4f), we found that in DK, both WT and NWT sites are located in pixels with an average HFI of around 14.9, implying there is human influence, but not as high as in urban areas (values towards 50). In AT, there is a difference in the median HFI of around 12.3 for NWT and 18.7 for WT sites, indicating that WPP sites are located on land with higher than average human influence. This is a consequence of AT being partly covered by the Alps, where low human influence prevails and where almost no wind turbines are installed. Since DK is a country without any significant elevation (Fig. 4g), most pixels are located between zero to 100 m above sea level. In AT, most WPP sites are located in the East, the flattest part of the country. This explains the high number of pixels at around 200 m above sea level. In addition, we observe some outliers at higher elevations which can be explained by wind parks in more mountainous regions, such as the previously mentioned Tauernwindpark. Only a marginal share of land is under nature conservation (Fig. 4h). As expected, NWT sites have a higher proportion of conservation areas compared with WT sites.

### Wind power deployment scenarios

**Fig. 5:**
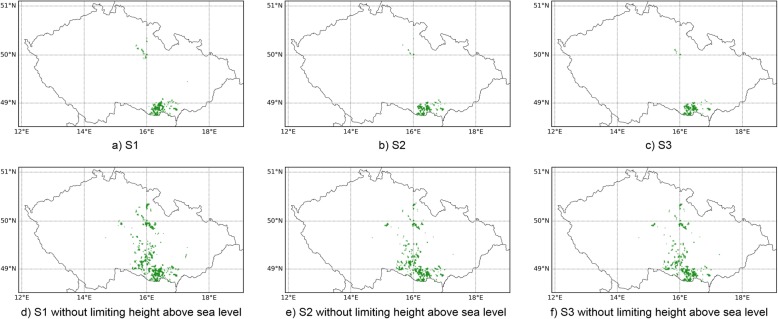
Allocation of the potential WPP sites in CZ in all calculated scenarios

The quartiles in the distribution of the site characteristics can be derived from the boxplots shown in the previous section (Fig. 4). They were used to generate wind power deployment scenarios for CZ. We estimated the potential land available for WPP twice for each of the three scenarios—with and without applying height above sea level as filtering criterion as listed in Table 2. In scenario S1, we used the observed first and third quartile of the respective characteristic in AT as a filtering criterion to identify eligible WPP. Thus, in scenario S1, we identified 543 km^2^ of land available for potential WPP deployment. The resulting potential allocation of WPP is shown on the map in Fig. 5a. There is a clear concentration in the southern part of CZ. When the average historically observed capacity density per pixel in AT (4.79 MW km^−2^) is used to estimate total potentials, a maximum of 2601 MW of WPP capacity is identified in CZ. In contrast, when using the DK thresholds as filter criteria in scenario S2, the area of available sites is reduced to 421 km^2^ and the WPP potential decreases to only 741 MW (Fig. 5b). This is a consequence of the much lower average capacity density observed in DK (1.76 MW km^−2^), and less land in CZ corresponding to DK site characteristics. In a mixed scenario S3, we did not distinguish between the origin of the observed characteristics, i.e., we mix characteristics in AT and DK. This resulted in an area of 409 km^2^ and a potential of 941 MW (based on a mean capacity density of 2.30 MW km^−2^) visualized in Fig. 5c.

**Table 2:** Results showing the suitable area in km^2^ for WPP installation in CZ

Scenario (source from where characteristics are derived)	S1 (*AT*)	S2 (*DK*)	S3 (mixed (AT and DK))
Standard	543 km^2^	421 km^2^	409 km^2^
Without limiting height above sea level	1370 km^2^	1105 km^2^	1032 km^2^

In alternative scenario runs, we did not restrict the height above sea level. In this case, the potential sites increase significantly to 1370 km^2^ (6562 MW) in the AT scenario S1, 1105 km^2^ (1945 MW) in the DK scenario S2, and 1032 km^2^ (2374 MW) in the mixed scenario S3 (Fig. 5d–f).

### Sensitivity analysis

In preliminary calculations, we found a high sensitivity of the average capacity density when excluding pixels with very low densities. In other words, when introducing a minimum threshold greater than 0.6 MW km^−2^ of WPP density per pixel, we observed a significant increase of the average WPP density in DK (Figure 9 in Appendix). This can be explained by a large number of outdated and low-capacity turbines in DK, such as the Vestas V17-75, Vestas V27-225, Bonus B31/300, or Micon M750-400 dating back to the 1990s. The average density was increasing from 1.76 MW km^− 2^ (no minimum density threshold applied) to 2.75 MW km^−2^ (density threshold greater than 0.6 MW km^−2^ applied). Applying this larger capacity density, our capacity estimates in the DK scenarios would therefore increase by 56%. For AT, the installed WPP densities did not change significantly because the data set did not feature such a large number of low-generation WPP.

**Fig. 6:**
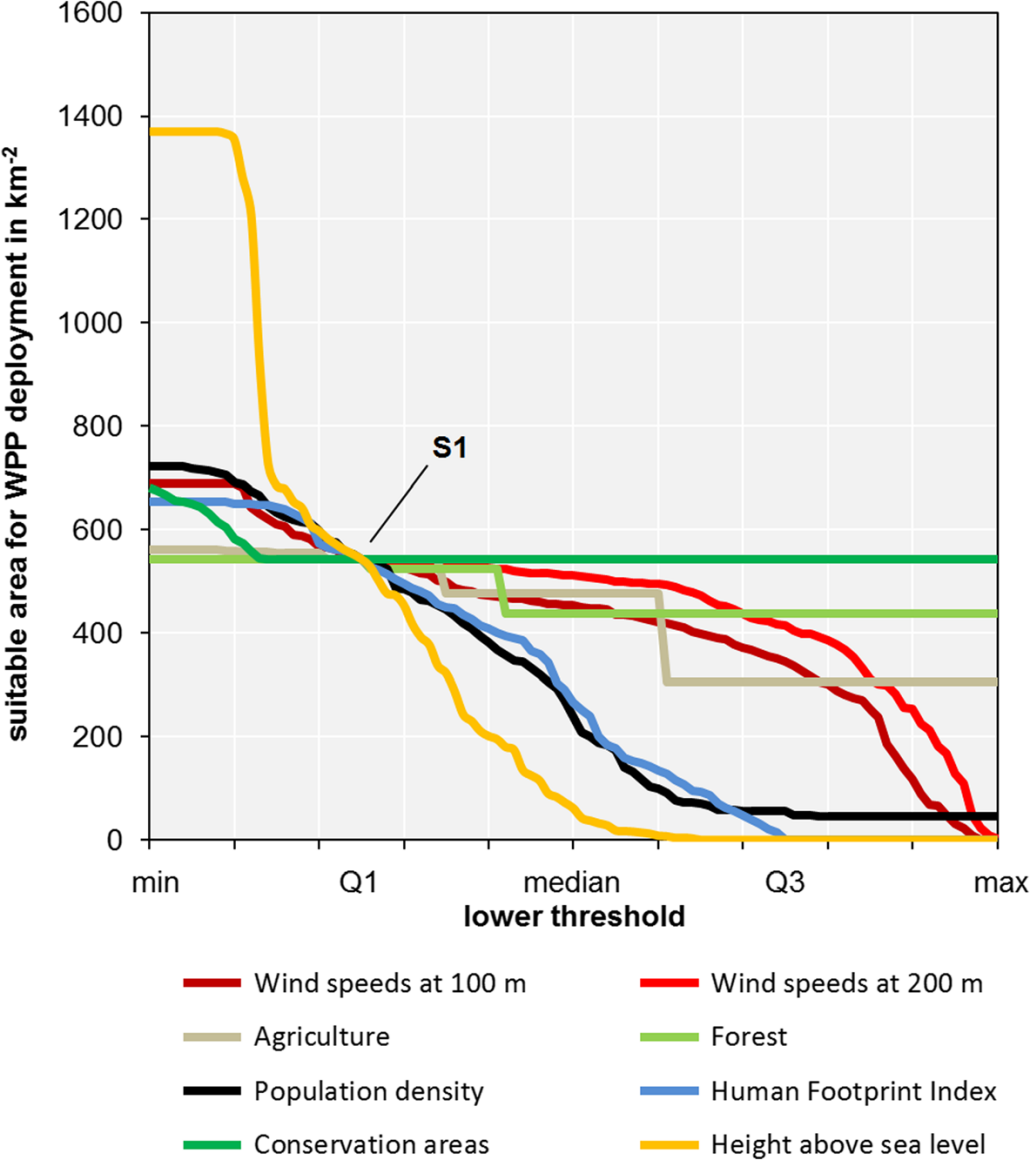
Sensitivity analysis in scenario S1 altering the lower thresholds for a single-selection criterion when the remaining selection criteria are kept unchanged

**Fig. 7:**
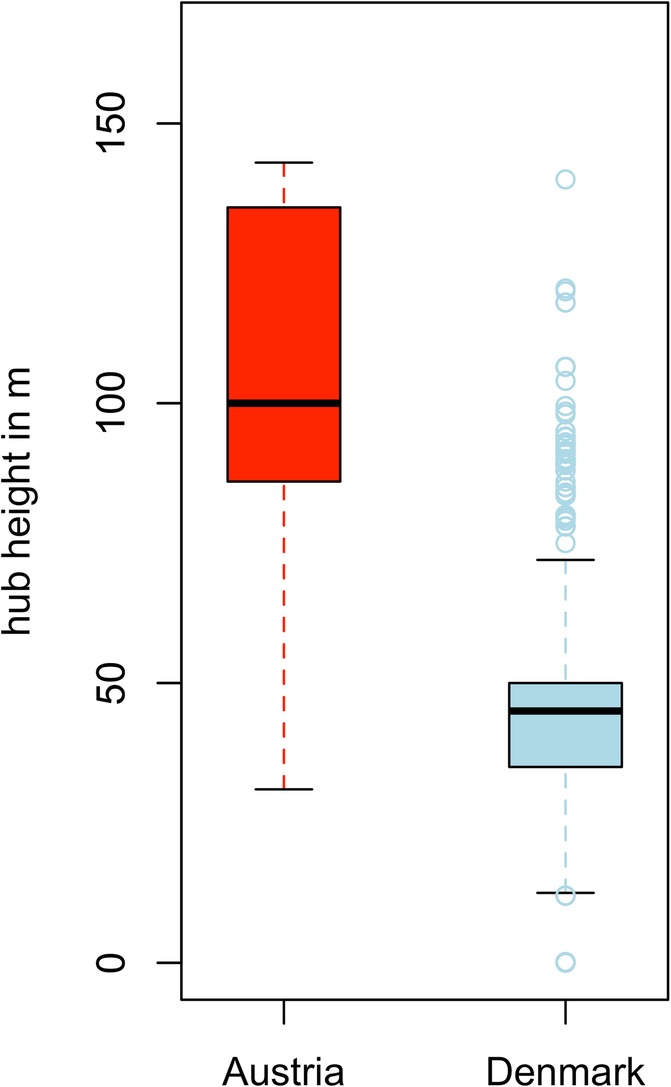
Rotor diameter of WPP in AT and DK in m

**Fig. 8:**
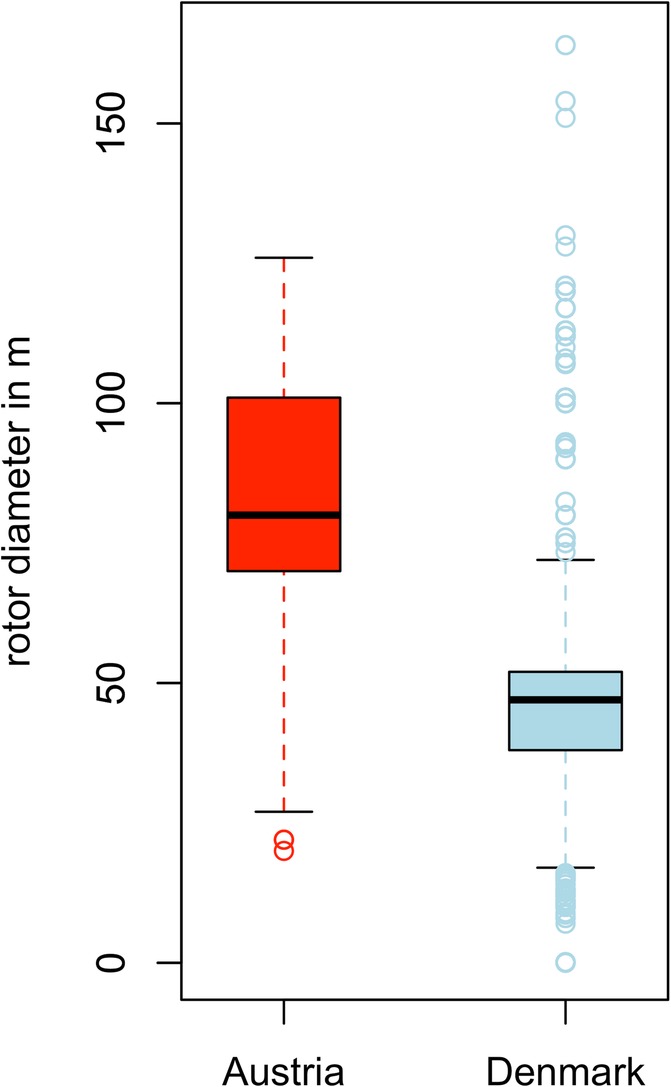
Hub height of WPP in AT and DK in m

**Fig. 9:**
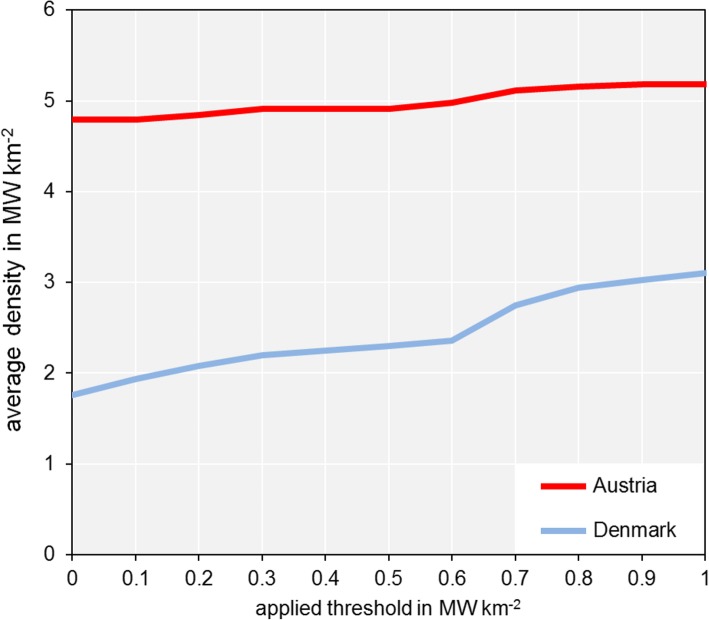
Average densities of WPP sites at different levels of minimum thresholds in MW km^−2^

In an additional analysis, we tested different assumptions for the filtering conditions. These can have a significant impact on the selection and number of possible WPP sites. In the sensitivity analysis, we gradually increased the lower limit of the selection criterion from the minimum of the observed distribution to the maximum, i.e., the range of possible values was decreased step by step, one parameter a time. Figure 6 shows the changes in new WPP capacity in scenario S1 when we modified the filter for a single-selection criterion while keeping the remaining selection criteria unchanged. On the *x*-axis, the different thresholds from minimum (left) to median (middle) to maximum (right) are plotted. In other words, the farther to the left, the less restrictive the filter conditions are; the farther to the right, the more restrictive the filter conditions are set. This explains the overall trend of higher possible capacities on the left. The intersection of all lines is the result of the previously presented scenario S1 in which the first quartile is set as minimum filter criterion. It can also be observed that the height above sea level has a significant influence on the results, when the minimum observed value is set as a filter. The HFI and the population density also clearly limit the possible WPP capacities when higher thresholds are applied. Regarding the agricultural lands, we observed a drastic decline in potential WPP capacities at around the 60% percentile. A smaller but still significant change can be observed for forests close to the 40% percentile. The presented land use data [32, 43] feature one of ten representative land use categories within a raster pixel and therefore result in significant leaps for forests and agricultural lands in Fig. 6. Conservation areas have hardly any influence on the results since the suitable area for WPP is significantly more restricted by the other factors used.

## Discussion

We compared our results with two existing European studies that report results for CZ. Ryberg et al. [11] use a top-down exclusion approach and determine an economic potential of up to 96 GW of wind power for CZ, about 15 times more than our most optimistic scenario (Austrian characteristics without restricting height above sea level). McKenna et al.’s [18] analysis shows an annual generation potential of 214 TWh, installed on an area for WPP of around 12,800 km^−2^ for CZ. At an assumed capacity factor of 26% [55], this is 14 times higher than the potential in our most optimistic scenario in terms of generation and about 9 times higher in terms of land availability. This shows the first strength and first limitation of our approach: it gives a very conservative estimate on the wind power potential for a region. However, it may be, at least in the short-term, much better aligned with the realizable potential than the top-down estimates cited above. With respect to the density of wind power installations, our empirical data shows comparable densities as reported in a paper by Miller et al. [27]. In DK, we observe 1.76 MW km^−2^ and in AT 4.79 MW km^−2^, while Miller et al. [27] report 2.80 MW km^−2^ for the USA. The higher density in Austria compared with both the USA and Denmark is related to (i) the later deployment of wind power, meaning that old, low-capacity turbines have not been installed to a large extent and (ii) likely differing spacing conventions in the three countries.

Our approach has several limitations. First, we assume that the expansion pattern in different countries can be transferred to a third country. A range of conditions, ranging from the implementation process of wind projects, public trust in general, and other factors influencing social acceptance and regulation, may differ between regions [25], and thus make our approach invalid. We assume that the current deployment patterns in the reference countries will remain stable. However, future deployment in the reference countries could yield higher wind power densities at new locations. Our analysis can, however, easily be repeated after new wind power plants have been built. Second, the selection of the site characteristics influences obtained results. We used a limited number of site characteristics. Possible extensions include information on road map data, since the installation and servicing of the turbines requires specific infrastructure nearby, information on the distance to the transmission grid, as grid connections are costly, and information on economic activities that may benefit or suffer from the deployment of wind turbines, e.g., tourism. A better understanding of how the wind turbines are spatially auto-correlated will also improve results, as the potential occurrence of spatial clustering in a country could be better simulated. Third, DK has different conditions for deploying wind power than CZ due to the possibility to build offshore WPP in the North and Baltic Sea. This could drastically reduce pressure on land for onshore WPP, as large-scale offshore wind parks can be built instead. Until 2022, DK has proposed to expand its offshore wind capacities by 1.35 GW with three main projects [56]. Fourth, the installed capacities for AT in this paper are slightly underrepresented compared with the numbers from today. The data set which was accessible for this analysis featured turbines with a total capacity of 2295 MW which is equivalent to the level of 2015. At the end of 2017, though a total of 2840 MW of wind power were installed in AT according to the Austrian Wind Energy Association [57]—an increase of 23%. Fifth, technological developments and rotor growth will lead to improved wind turbines and to potential higher capacity densities [58, 59]. This will change the use of land suitable and potentially available for WPP. There are more powerful WPP necessary to generate the same amount of electricity on smaller sites. This can also reduce the pressure on available land. Further research is required to investigate the effects of future technological improvements on land availability for WPP.

## Conclusions

Land availability assessments are crucial for understanding the limits to the expansion of wind power plants, and renewable generation technologies in general. We propose a new, conservative method to complement existing studies on land availability for wind power generation by deriving criteria for eligible land from observed characteristics of wind power plant sites. To the best of our knowledge, this presented approach is the first time conducted in the context of wind power potential assessments. We calculated the density of installed wind turbines which is on average higher in Austria with 4.79 MW km^−2^ compared with Denmark with 1.76 MW km^−2^. As expected, the overall mean wind speeds in 100 m and 200 m above ground level are higher than average for locations where wind turbines are installed. Most wind turbines are deployed in areas with high shares of agriculture (on average 86%) and only a minor share of forests (on average 7%). The Human Footprint Index shows that wind turbines in Austria are installed in areas with higher human impact compared with the country’s average. However, this is not the case for Denmark where no significant difference was found.

Regarding the availability of land for wind power installation, our results are an order of magnitude lower than the potentials in existing studies. This points to high levels of uncertainty regarding the future potential for wind power generation. In particular, our results showed that Danish site characteristics in scenario S2 limit the area of available sites significantly more than in scenario S1 where Austrian thresholds are applied. The main limiting factors in Czechia are population density, human impact on land, prevalent wind speeds, and the height above sea level. Conservation areas such as national parks and Natura 2000 areas have only a marginal impact. The data sets presented here can be used as a reference for the calculation of future wind power potentials for other regions in Europe. While the 1-km resolution provides insights in land use at wind power sites and wind power densities, detailed on-site assessments with higher resolution than 1 km may further improve our results. Additionally, the analysis can be extended by testing new countries as source for the description of characteristics. The presented results could be used as an additional input for technical and economic assessments of wind power generation, contributing to overcome shortcomings of existing studies.

## Data Availability

The input data sources D1 and D3-9 as described in Table 1 are available for open-access download. The data set D2 was kindly provided by the Austrian Wind Energy Association [29] and is available upon request. The aggregated raster pixel data sets with 1-km spatial resolution for the research areas Austria, Denmark, and the Czech Republic are provided by Nitsch et al. [60]. The Python code used in this assessment is made available online by Nitsch [53].

## Abbreviations

AT: Austria
_*c*_: Country in the filtering process (AT, DK, mixed)
CZ: Czechia
_*d*_: Data set used in the filtering process (D3-D9)
D1-D9: Data sets as listed in Table 1
DK: Denmark
GIS: Geographic information system
HFI: Human Footprint Index
_*i*_: Index of pixels with at least one WPP
_*j*_: Index of pixels without WPP
*L*: Lower filter condition for the selection of eligible pixels
NWT: Pixels without any wind power plant
*P*: Selection criterion for the selection of eligible pixels
S1: Scenario with thresholds derived from observed data in Austria
S2: Scenario with thresholds derived from observed data in Denmark
S3: Scenario with thresholds derived from observed data in Austria and Denmark
*U*: Upper filter condition for the selection of eligible pixels
*Q*_0.25_: First quartile
*Q*_0.75_: Third quartile
WPP: Wind power plant
WT: Pixels with at least one wind power plant
